# Integration of Polymyxin-B Hemoadsorption Device into a CRRT Circuit for Endotoxic Septic Shock in a Child: A Case Report

**DOI:** 10.3390/pediatric18020037

**Published:** 2026-03-04

**Authors:** Giovanni Ceschia, Germana Longo, Jose M. Igeno San Miguel, Marco Daverio, Enrico Vidal

**Affiliations:** 1 Pediatric Nephrology Unit, Department of Woman’s and Child’s Health, University-Hospital of Padova, 35128 Padova, Italy; germana.longo@aopd.veneto.it (G.L.); jose.igeno@aopd.veneto.it (J.M.I.S.M.); enrico.vidal@aopd.veneto.it (E.V.); 2 Pediatric Critical Care Unit, Department of Woman’s and Child’s Health, University-Hospital of Padova, 35128 Padova, Italy; marco.daverio@aopd.veneto.it; 3 Department of Medicine (DMED), University of Udine, 33100 Udine, Italy; 4 Institute of Pediatric Research “Città della Speranza”, 35127 Padova, Italy

**Keywords:** pediatric hemoadsorption, continuous renal replacement therapy, acute kidney injury, pediatric septic shock, extracorporeal endotoxin removal

## Abstract

**Introduction**: Endotoxin-mediated septic shock is a life-threatening condition characterized by systemic inflammation and hemodynamic instability. While Polymyxin-B hemoadsorption (Toraymyxin^®^) is well-studied in adults, its use in pediatric patients remains less explored and requires modified approaches to minimize invasiveness and complications. **Case Presentation**: We report a 9-year-old boy (25 kg) with endotoxin-mediated septic shock due to Klebsiella pneumoniae, who developed oliguric acute kidney injury requiring continuous renal replacement therapy (CRRT). On Day 4, worsening conditions prompted the initiation of Toraymyxin^®^ treatment, directly integrated into the ongoing CRRT circuit. This approach minimized extracorporeal volume expansion, avoided circuit replacement, and was complication-free. The patient improved rapidly, allowing CRRT discontinuation and transfer to the ward within 28 days. **Conclusions**: This case highlights the feasibility, safety, and potential benefits of integrating the Toraymyxin^®^ cartridge into an ongoing CRRT circuit in pediatric septic shock, minimizing extracorporeal volume, avoiding additional vascular access, and supporting hemodynamic stabilization.

## 1. Introduction

Endotoxin-mediated septic shock is a life-threatening condition characterized by systemic inflammation and hemodynamic instability, primarily driven by high endotoxin concentrations in the bloodstream. Endotoxin activates the immune system, triggering the release of pro-inflammatory cytokines, endothelial dysfunction, and impaired tissue perfusion. These mechanisms exacerbate disease severity and can rapidly progress to multiple organ failure [1].

Extracorporeal endotoxin removal via hemoadsorption has recently gained attention, particularly with the Polymyxin-B hemoadsorption device (Toraymyxin^®^, Toray Medical, Tokyo, Japan). This device utilizes polymyxin B immobilized on polystyrene fibers to selectively remove endotoxin, offering a promising therapeutic approach for endotoxin-mediated septic shock. In Europe, two device sizes are available: the larger PMX-20R^®^ (135 mL priming volume) and the smaller PMX-05R^®^ (40 mL priming volume). The efficacy of the Toraymyxin^®^ PMX-20R has been evaluated in adults through multiple studies [2,3,4]. One randomized controlled trial (RCT) assessed 64 patients with Gram-negative septic shock: 34 patients received hemoadsorption with Toraymyxin^®^ PMX-20R in addition to conventional therapy, while 30 underwent conventional therapy only. The study demonstrated improved 28-day survival in the hemoadsorption group [2]. Another large-scale trial involving 450 adults with an endotoxin activity level (EA) ≥0.60 units found no overall survival benefit [3]. However, a post hoc analysis identified a subgroup with intermediate EA levels (0.60–0.89) who exhibited reduced 28-day mortality [4]. An additional RCT is currently recruiting patients with intermediate EA levels to validate these findings (NCT03901807).

In contrast, evidence on the use of Toraymyxin^®^ in pediatric populations remains limited, consisting of case reports and small observational studies [5,6,7,8]. The largest study to date was a prospective cohort study involving 15 children (>6 kg) who developed endotoxic septic shock (EA >0.6 units) following cardiac surgery. Treatment with the PMX-05R cartridge resulted in clinical improvement, reduction in EA levels, and an 80% 28-day survival rate, with no reported safety concerns. In this study, hemoadsorption was performed via hemoperfusion (without renal replacement therapy circuits) in 13 patients, while in the remaining two cases, it was integrated into an intermittent hemodialysis circuit [6].

Here, we report, to our knowledge, the first case of a child with septic shock and elevated EA levels in whom the Toraymyxin^®^ PMX-20R cartridge was directly integrated into an ongoing continuous renal replacement therapy (CRRT) circuit. This report focuses on the technical aspects of the integration, highlighting practical considerations and challenges encountered when incorporating the hemoadsorption device into the CRRT system.

## 2. Case Presentation

We present the case of a 9-year-old male weighing 25 kg, diagnosed with B-cell lymphoblastic leukemia undergoing chemotherapy for central nervous system relapse. He was admitted to the pediatric intensive care unit (PICU) for fluid-refractory septic shock. At admission (Day 1), the patient was neutropenic, presenting with abdominal pain and diarrhea. Blood cultures were positive for Klebsiella pneumoniae. Broad-spectrum antimicrobial therapy was initiated upon admission.

In the early hours after admission, the patient’s condition deteriorated, requiring invasive mechanical ventilation and escalation of hemodynamic support including initiation of stress-dose steroids. On Day 2, he developed oliguric acute kidney injury (AKI) with severe fluid accumulation, requiring renal replacement therapy. At 22:00 on Day 2, CRRT with regional citrate anticoagulation was initiated using the PrisMax^®^ monitor (Baxter International Inc., Deerfield, IL, USA) with an ST60^®^ set (Baxter International Inc.). CRRT was initiated with good hemodynamic tolerance (treatment parameters shown in Table 1).

On Day 4, clinical deterioration necessitated increased vasopressor support. The EA returned a value of 1.07, indicative of severe endotoxemia; therefore, extracorporeal endotoxin removal therapy was initiated using the Toraymyxin^®^ PMX-20R cartridge as rescue therapy. While the smaller PMX-05R cartridge was available in our institution, we opted for the PMX-20R, recommended for patients ≥25 kg, to ensure adequate adsorption capacity.

Baseline serum interleukin-6 (IL-6) levels, measured prior to cartridge connection, were markedly elevated (Table 2).

**Figure 1:**
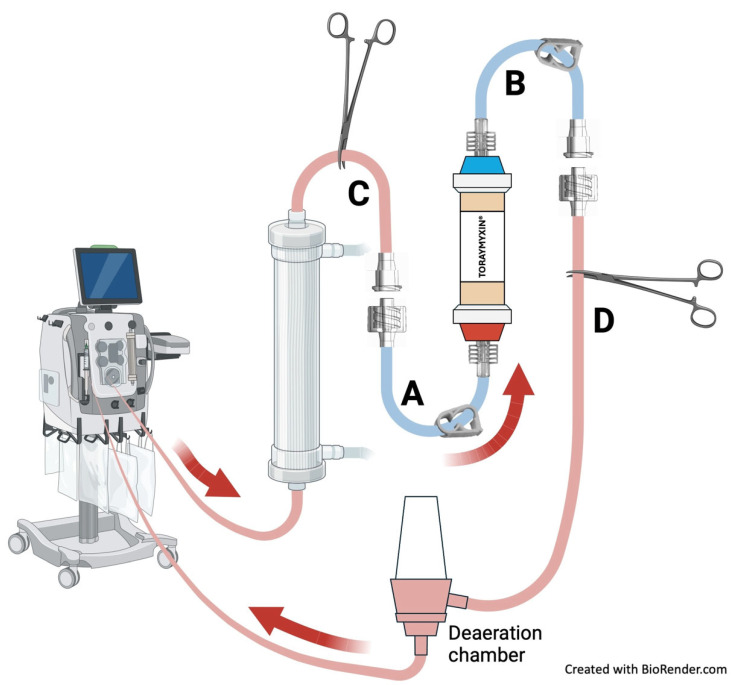
In-line connection and disconnection of the Toraymyxin^®^ PMX-20R cartridge to the ongoing CRRT circuit. After priming, the cartridge had an inlet (male, “A”) and outlet (female, “B”), both clamped. The CRRT circuit was paused, and tubing clamped after the hemofilter (“C”) and before the air chamber (“D”). The segment between clamps was disconnected, and the cartridge was integrated in-line by connecting “A” to “C” and “B” to “D.” Clamps were removed, and CRRT resumed. For disconnection, the circuit was paused, tubing clamped at points “A,” “B,” “C,” and “D,” connections “A–C” and “B–D” released, the cartridge discarded with “A” and “B” attached, and the original CRRT circuit re-established. Red arrows show the direction of blood flow through the circuit.

The cartridge was integrated in-line into the ongoing CRRT circuit for a 2-h treatment session (Figure 1). Connection and subsequent removal were performed after brief pauses of the circuit, without replacing the hemofilter set. No complications occurred during connection or disconnection (including blood leak, clotting, air entrainment, or contamination), and CRRT was continued using the original circuit. Citrate dose was increased at cartridge initiation (from 2.7 to 3 mmol/L) to minimize clotting risk and subsequently adjusted according to standard protocol.

Within a few hours post-treatment, the patient’s hemodynamics began to improve. By the next morning, the Vasopressor-Inotropic Score (VIS) had decreased, alongside reductions in the Pediatric Logistic Organ Dysfunction 2 (PELOD-2) score and Phoenix Sepsis Score (Table 2).

On Day 6, the patient remained in critical condition under significant inotropic support. EA was still elevated (0.91), prompting a second Toraymyxin^®^ PMX-20R session. The cartridge was again safely integrated in-line using the same strategy as before, without replacing the hemofilter set. The following morning, hemodynamics improved, with only one vasopressor required at a low dose. Serum IL-6 concentrations on Day 7 showed a significant decrease (Table 2). CRRT was discontinued on Day 11. The patient’s clinical condition continued to improve, and he was transferred to the general ward on Day 24.

### Technical Specifications of Toraymyxin^®^ PMX-20R Integration into Ongoing CRRT Circuit

To provide technical insights, we describe the step-by-step procedure for connecting and disconnecting the Toraymyxin^®^ cartridge to the ongoing CRRT circuit (Figure 1, reference points A–D):

(1) The Toraymyxin® cartridge was primed according to the manufacturer’s instructions. Specifically, it was flushed with 4 L of 0.9% NaCl. After priming, the cartridge had two connected tubes: one for the inlet (male end, “A”) and one for the outlet (female end, “B”). Both lines were clamped.
(2) The CRRT treatment was paused.
(3) The CRRT tubing was clamped after the hemofilter (“C”) and before the air chamber (“D”).
(4) The tubing system between the clamps was disconnected.
(5) The cartridge inlet tube (male) was connected to the female end of the tubing coming from the hemofilter.
(6) The cartridge outlet tube (female) was connected to the tubing section leading to the air chamber.
(7) Clamps were removed, and the treatment was restarted.
(8) After 2 h of treatment, the cartridge was disconnected, and CRRT was resumed. The disconnection procedure was similar to the connection process, involving pausing the treatment, clamping the tubing at points “A”, “B”, “C”, and “D”, disconnecting “A” from “C” and “B” from “D”, discarding the cartridge with “A” and “B” connected, and reconnecting “C” to “D.”

## 3. Discussion

Polymyxin-B hemoadsorption is increasingly used in intensive care units as an adjuvant therapy for endotoxin-mediated septic shock. In adults, larger blood volume and easier central venous access simplify extracorporeal treatments, explaining the manufacturer’s recommendation for standalone hemoperfusion circuits. In pediatric patients, smaller blood volume and limited venous access make standalone setups often impractical or unsafe. Placing a dedicated large-bore central line or adding a parallel hemoperfusion circuit alongside ongoing CRRT is frequently unfeasible.

Only one published study describes Toraymyxin^®^ connected to another extracorporeal circuit in children; however, that involved intermittent hemodialysis, a short-duration treatment similar to standard hemoperfusion. To our knowledge, this is the first report describing the use of Toraymyxin^®^ connected to an ongoing CRRT circuit in a pediatric patient.

Our patient had an estimated total blood volume of approximately 1.8 L, meaning the extracorporeal volume (ECV) should ideally remain below 180 mL (10% of total blood volume) to minimize the risk of hemodynamic instability. The ST60^®^ set already in use had an ECV of 97 mL, leaving a theoretical margin of 83 mL. While the smaller PMX-05R cartridge could have fit, we opted for the PMX-20R, recommended for patients ≥25 kg, to ensure adequate adsorption capacity.

Different technical configurations for hemoadsorption were evaluated. A standalone hemoperfusion setup would have required an additional large-bore vascular access and a dedicated extracorporeal circuit, increasing procedural invasiveness. A parallel circuit connected to the existing catheter would have avoided new vascular access but still required a complete additional circuit, resulting in a higher total ECV compared with inline integration. Inline integration into the CRRT circuit therefore represented the most advantageous option. However, the conventional strategy of stopping CRRT and restarting treatment with a newly assembled circuit including the hemoadsorption cartridge would have exposed the patient to the full combined ECV of the CRRT set and cartridge simultaneously, markedly exceeding the safety threshold.

For this reason, we connected the PMX-20R in-line to the ongoing CRRT circuit already tolerated by the patient. By briefly pausing the circuit and inserting only the cartridge, the incremental ECV (147 mL) remained within the safety limit, minimizing hemodynamic stress. The same strategy allowed safe cartridge removal and subsequent reintegration for the second session without replacing the CRRT circuit. This strategy allowed the procedure to be performed without hemodynamic compromise or the need for blood priming, while providing optimal adsorption capacity for endotoxin removal.

The safety of this approach relies on careful procedural planning. Both connection and disconnection involve briefly opening a blood-filled circuit, which could theoretically increase the risk of infection, air entrainment, or clot formation. In our case, these risks were mitigated by strict sterile technique, rapid execution by trained personnel, and the use of regional citrate anticoagulation to reduce clotting during temporary blood stagnation.

Although not intended to assess efficacy, a brief descriptive overview of clinical scores and biomarkers was included for contextual purposes. After the first hemoadsorption session, VIS, PELOD-2, and Phoenix Sepsis Score decreased, while after the second session only VIS further improved, with organ dysfunction scores remaining stable. EA showed a partial reduction after the first session but was not available after the second session. IL-6 levels decreased between the pre–first and post–second session measurements; however, IL-6 was assessed only at these two time points, precluding pre–post evaluation of individual sessions. As IL-6 is not expected to be directly removed by Toraymyxin^®^, this reduction may reflect the natural clinical evolution of an improving septic shock episode; nonetheless, a contributory effect mediated by endotoxin removal and downstream attenuation of the cytokine cascade cannot be excluded. Given the single-case design, incomplete biomarker kinetics, and absence of a control condition, no conclusions regarding efficacy can be drawn; nevertheless, the rapid overall clinical improvement temporally associated with the intervention does not exclude a contributory role of hemoadsorption.

This case demonstrates the technical feasibility, safety, and reproducibility of inline integration of polymyxin-B hemoadsorption into an ongoing CRRT circuit in a pediatric patient. The described approach highlights practical strategies to optimize extracorporeal volume management, avoid additional vascular access, and allow cartridge insertion and removal without circuit replacement. These considerations may be particularly relevant in pediatric settings where blood volume constraints, limited vascular access, or the absence of dedicated pediatric cartridges render standalone hemoperfusion impractical.

## Figures and Tables

**Table 1:** Specifications of the CRRT treatment with ST60^®^ set alone and during PMX-20R connection.

Parameter	ST60^®^ Set Alone	ST60^®^ Set + PMX-20R
BFR (mL/min)	100	100
Citrate dose (mmol/L)	2.7	3.0
Effluent dose (mL/h/1.73 m^2^)	2000	2400
ECV (mL)	97	244 (97 + 135 * + 12 **)

BFR—blood flow rate; ECV—extracorporeal volume; * ECV of Toraymyxin^®^ PMX-20R; ** ECV of tube connectors.

**Table 2:** Clinical and biochemical parameters before and after PMX-20R treatment.

Variable	Day 4 8:00 (Pre)	Day 4 16:00	Day 5 8:00 (Post)	Day 6 8:00 (Pre)	Day 6 16:00	Day 7 8:00 (Post)
VIS	60	**PMX**	28	12	**PMX**	7
IL-6 (ng/L)	50,000		n.a.	n.a.		1268
EA (units)	1.07		0.98	0.91		n.a.
PELOD-2	10		9	9		9
Phoenix Sepsis Score	8		6	6		6
Fluid accumulation (%)	14		11	9		7

VIS—vasoactive inotropic score; IL-6—interleukin 6; EA—endotoxin activity; PELOD-2—Paediatric Logistic Organ Dysfunction 2.

## Data Availability

The original contributions presented in this study are included in the article. Further inquiries can be directed to the corresponding author.
